# Family resilience, dyadic coping, and health-related quality of life among primary caregivers of hospitalized older adults with multimorbidity in Xinjiang, China: a latent profile and mediation analysis

**DOI:** 10.3389/fpubh.2026.1916251

**Published:** 2026-10-08

**Authors:** Weilong Wang, Runjia Zhang, Xuexia Yang, Yongle Pei, Zhengwei Luo, Xiaoxuan He, Shufeng Ren, Qianqian Jia, Jingwen Cai, Bo Zhang, Liang Liu, Wen Cai

**Affiliations:** 1 Department of Nursing, Xinjiang Medical University, Urumqi, Xinjiang, China; 2 The Fourth Affiliated Hospital of Xinjiang Medical University, Urumqi, Xinjiang, China; 3 Institute of Health Management, Xinjiang Medical University, Urumqi, Xinjiang, China

**Keywords:** caregiver, dyadic coping, family resilience, health-related quality of life, latent profile analysis, mediation analysis, multimorbidity, older adults

## Abstract

**Background:**

Older adults with multiple chronic conditions impose a heavy caregiving burden on their families. However, little is known about how family resilience and dyadic coping are associated with caregivers' health-related quality of life (HRQoL). This study aimed to identify latent profiles of family resilience among primary family caregivers of hospitalized older adults with multimorbidity and to examine whether dyadic coping was indirectly associated with the relationship between family resilience and caregiver HRQoL within a hypothesized mediation model.

**Methods:**

From October 2025 to June 2026, 572 primary family caregivers of older inpatients with multimorbidity were recruited from three tertiary hospitals in Xinjiang, China. Participants completed the Chinese version of the Family Resilience Assessment Scale (FRAS-C), the Dyadic Coping Inventory (DCI), and the 12-item Short Form Health Survey (SF-12). Latent profile analysis (LPA) was used to identify distinct profiles of family resilience. Univariate and multivariate logistic regression analyses were performed to determine factors distinguishing these profiles. Hypothesized mediation models using the bootstrap method were tested to estimate statistical indirect associations, with caregiver age, education level, monthly income, daily caregiving hours, and self-rated health as covariates.

**Results:**

Three distinct family resilience profiles were identified: low (33.9%), middle (41.6%), and high (24.5%) family resilience. Using the low resilience group as the reference, statistically significant indirect associations through dyadic coping were observed between moderate resilience and physical HRQoL (indirect effect = 0.5825, 95% *CI*: 0.3317–0.8801), moderate resilience and mental HRQoL (indirect effect = 0.5103, 95% *CI*: 0.2701–0.7663), high resilience and physical HRQoL (indirect effect = 1.2105, 95% *CI*: 0.8039–1.7050), and high resilience and mental HRQoL (indirect effect = 1.0605, 95% *CI*: 0.6858–1.5003). Multivariate logistic regression showed that caregiver education level, monthly income, daily caregiving hours, self-rated health, and caregiver age were independent factors distinguishing the resilience profiles. Stratified analyses further showed that the positive associations between dyadic coping and HRQoL were consistent across spousal, child, and other caregiver subgroups.

**Discussion:**

Findings were consistent with the hypothesized mediation model, showing indirect associations between family resilience and both physical and mental HRQoL through dyadic coping. Strengthening family resilience and promoting dyadic coping may be a promising approach to supporting caregivers' health outcomes.

## 1 Introduction

In recent decades, the prevalence of multimorbidity—the coexistence of two or more chronic conditions in an individual—has risen significantly due to global population aging and increasing life expectancy (1). Multi-country studies indicate that the self-reported prevalence of multimorbidity increased from 12.7% in 2001 to 16.2% in 2011 (2). Furthermore, developing countries are witnessing a rapid rise in multimorbidity rates, driven by longer life expectancy and often constrained public health resources (3, 4). For instance, a study utilizing the China Health and Retirement Longitudinal Survey (CHARLS) database, which included 10,479 adults aged 60 years and older, revealed that the prevalence of multimorbidity among older Chinese adults reached as high as 65.6% (5).

Health-related quality of life (HRQoL) serves as a fundamental indicator of caregivers‘ overall well-being, incorporating both physical and mental health dimensions (6). Family caregivers of older adults with multimorbidity frequently encounter substantial caregiving responsibilities, resulting in a notable decline in their HRQoL (7). Diminished HRQoL among caregivers adversely impacts their health and compromises the quality of care provided, subsequently affecting patients' disease outcomes (8). Consequently, it is crucial to identify protective factors that enhance caregiver HRQoL and to comprehend the mechanisms underlying these factors in order to formulate sustainable family care strategies (9).

Family resilience denotes the capacity of a family system to adapt positively to adversity through mechanisms such as effective communication, collaborative problem-solving, and the mobilization of social resources (10). According to Walsh's family resilience theory, families exhibiting high resilience can reframe the meaning of adversity, sustain cohesion, and actively seek external support, thus mitigating the negative psychological impacts of caregiving burden (11). A recent systematic review revealed that greater family resilience is significantly correlated with reduced caregiver burden, diminished psychological distress, and improved quality of life (12). Nevertheless, the specific associations of family resilience with caregiver HRQoL and the underlying mechanisms remain unclear (9).

Dyadic coping—the process through which caregivers and patients communicate about stressful events and engage in joint coping strategies—may be an intervening variable in this relationship (13, 14). When caregivers exhibit low levels of family resilience, dyadic coping among family members may also be low, and this may be associated with poorer communication and inadequate support, which in turn may be associated with poorer caregiver HRQoL. Conversely, as a positive joint coping mechanism, dyadic coping may be one pathway through which family resilience is associated with HRQoL and may assist caregivers in better managing caregiving stress. According to Bodenmann's systemic transactional model, dyadic coping reframes stress from “my burden” to “our problem,” thereby fostering mutual support and alleviating caregivers' feelings of isolation (13). Research involving maintenance hemodialysis patients and their caregivers has shown that dyadic coping is associated with the quality of life for both patients and caregivers (15). Similarly, studies on patient-spouse caregiver dyads with coronary heart disease have examined resilience as an intervening variable in the relationship between dyadic coping and post-traumatic growth. However, the indirect association between family resilience and HRQoL through dyadic coping among primary caregivers of hospitalized older adults with multimorbidity remains insufficiently explored (16). Therefore, we propose that family resilience may be indirectly associated with HRQoL through dyadic coping within a hypothesized mediation model.

Previous studies have predominantly employed variable-centered approaches, which overlook the heterogeneity present among various clinical subgroups of patients (17). In contrast, person-centered latent profile analysis (LPA) effectively identifies heterogeneous subgroups based on individuals' response patterns across multiple dimensions (18). In recent years, LPA has been utilized to investigate heterogeneity in family resilience across diverse populations (19). However, no study has specifically applied LPA to characterize the heterogeneity of family resilience among primary caregivers of hospitalized older adults with multimorbidity (20).

Therefore, this study hypothesized that: (1) distinct heterogeneous profiles of family resilience among primary caregivers of hospitalized older adults with multimorbidity would be identified using LPA; (2) differences in patterns of dyadic coping and HRQoL across family resilience profiles would be compared; and (3) based on the profile findings, the indirect association between family resilience profiles and HRQoL through dyadic coping would be examined. The results will provide a reference for developing individualized, stratified psychosocial intervention strategies (20).

Based on previous research, we proposed the following hypotheses:

H1: LPA will identify distinct latent subtypes of family resilience among primary caregivers of hospitalized older adults with multimorbidity.
H2: Family resilience profiles would be indirectly associated with HRQoL through dyadic coping within a hypothesized mediation model.

## 2 Methods

### 2.1 Participants and recruitment

Using convenience sampling, we recruited primary caregivers of hospitalized older adults (aged ≥ 60 years) with multimorbidity from the departments of Geriatrics, Cardiovascular Medicine, Respiratory Medicine, and Endocrinology in three tertiary hospitals in Urumqi between October 2025 and June 2026 (21). Multimorbidity was defined as having two or more chronic conditions based on the Charlson Comorbidity Index (CCI) (22). Patient medical records were reviewed only to confirm eligibility; CCI scores, number of chronic conditions, specific diagnoses, and medication information were not retained in the analytical dataset. All eligible caregivers had been the primary caregivers of the patients during the data collection period and had provided care for at least 1 week. Inclusion criteria were: (1) age ≥ 18 years; (2) identified as the patient's primary caregiver assuming main caregiving responsibility; (3) clear consciousness and voluntary participation. Exclusion criteria were: (1) severe cognitive impairment or mental illness; (2) paid caregivers; (3) refusal to participate.

A total of 593 questionnaires were distributed, and 572 valid questionnaires were returned, giving an effective response rate of 96.46%. Before the survey, the researchers explained the purpose and significance of the study to the caregivers. Written informed consent was obtained, and participants completed the questionnaires by themselves. For those with visual or writing difficulties, the researchers provided on-site assistance.

The present caregiver cohort was derived from the same parent protocol as our previously published patient-focused study (21), but the caregiver dataset was independent and not paired at the individual level with the patient dataset. The parent ethics protocol prospectively covered both patients and their family caregivers.

### 2.2 Sample size

The sample size required for LPA is influenced by several factors, including the number of indicators, the true number of classes, the distance between classes, and the model selection method. Simulation studies by Tein et al. (17) showed that a sample size exceeding 300 is sufficient to ensure stable and accurate classification when the number of indicators is moderate and class separation is acceptable. In this study, the 32 individual items of the Family Resilience Scale were used as LPA indicators, and the sample size of 572 met this requirement. Furthermore, Tein et al. (17) indicated that when the distance between classes is moderate, a sample size of 250 to 500 is sufficient for methods such as BIC, LMR, and BLRT to accurately identify the number of classes. Therefore, our sample size adequately supports stable LPA classification.

Regarding hypothesized mediation models, MacKinnon et al. (23) suggested that a sample size of 200 to 400 provides adequate statistical power for testing indirect effects using the bias-corrected bootstrap method. In summary, the 572 primary caregivers included in this study ensured sufficient statistical power for all analyses.

### 2.3 Instrument

#### 2.3.1 Demographic characteristics

Based on our research team's previous studies and clinical experience, demographic variables were divided into two categories: (1) sociodemographic characteristics, including age, sex, relationship with the patient, education level, and monthly household income; and (2) caregiving-related characteristics, including daily caregiving hours and self-rated health status.

#### 2.3.2 Measurement of family resilience

Family resilience was measured using the Family Resilience Assessment Scale-Chinese Version (FRAS-C) (24). This scale was originally developed by Sixbey based on Walsh's family resilience theory (25). It consists of 32 items divided into three dimensions: Family Communication and Problem Solving (FCPS, 27 items), Utilization of Social Resources (USR, 3 items), and Maintaining a Positive Attitude (MPO, 2 items). Each item is rated on a 4-point Likert scale (1 = strongly disagree, 4 = strongly agree). Total scores range from 32 to 128, with higher scores indicating higher levels of family resilience (24, 26). In the Chinese population, the Cronbach's α coefficient of this scale is 0.944, the split-half reliability is 0.807, and the test-retest reliability is 0.917. Chu and colleagues validated the FRAS-C among Chinese family caregivers, and the Cronbach's α values for the subscales ranged from 0.724 to 0.963 (24–26). In this study, the Cronbach's α of the scale was 0.847.

#### 2.3.3 Measurement of dyadic coping

Dyadic coping was measured using the Dyadic Coping Inventory (DCI). This inventory was developed by Bodenmann to assess supportive behaviors between partners when facing stress (14). The Chinese version of the DCI contains 37 items divided into six dimensions: Stress Communication (6 items), Supportive Dyadic Coping (10 items), Delegated Dyadic Coping (4 items), Common Dyadic Coping (5 items), Negative Dyadic Coping (8 items), and Evaluation of Dyadic Coping (2 items). The last two items form a separate evaluation dimension and are not included in the total score, consistent with the original scale design (13, 27). Each item is rated on a 5-point Likert scale (1 = very rarely, 5 = very frequently). Total scores range from 35 to 175, with higher scores indicating higher levels of dyadic coping. Xu et al. (28) validated this scale in 474 Chinese couples and confirmed its good structural validity and cross-cultural measurement invariance. In Chinese caregiver populations, the Cronbach's α of the total scale is 0.84, and the Cronbach's α values for the subscales range from 0.64 to 0.81 (28). In this study, the Cronbach's α of the scale was 0.812.

#### 2.3.4 Measurement of health-related quality of life

HRQoL was assessed using the 12-Item Short-Form Health Survey (SF-12). The SF-12 is a shortened version of the SF-36 and has been widely used in older adult and caregiver populations (6). The scale consists of 12 items and yields two dimension scores: the Physical Component Summary (PCS) and the Mental Component Summary (MCS).

In this study, the raw total scores of the PCS and MCS were computed by summing the respective items, rather than using the standard norm-based scoring algorithm. This approach was chosen based on the recommendation of Hagell et al. (29), who demonstrated that the standard SF-12 scoring algorithm is based on the problematic assumption that physical and mental health are uncorrelated, which can lead to distorted measurement and score interpretations. The raw sum score provides a simpler and more valid representation of physical and mental health. The PCS comprised six items (1–5, 8) and the MCS six items (6, 7, 9–12). Four items (1, 8–10) were reverse-scored. Raw sum scores were obtained by directly summing the reverse-scored items, yielding theoretical ranges of 6–26 for PCS, 6–30 for MCS, and 12–56 for the overall HRQoL score. An overall HRQoL score was computed as the sum of PCS and MCS. Higher scores are indicative of better quality of life. In the Chinese community-dwelling older adult population, the Cronbach's α of the SF-12 is 0.910, and the split-half reliability is 0.812, indicating good internal consistency (30). The SF-12 has also been widely used among caregivers; for example, among memory clinic caregivers, the Cronbach's α ranged from 0.781 to 0.785 (6, 30). In this study, the Cronbach's α of the scale was 0.723.

### 2.4 Statistical analysis

First, descriptive statistics were used to summarize the data. Continuous variables were expressed as mean ± standard deviation (M ± SD), and categorical variables as frequencies and percentages (*n*, %). Second, the Shapiro–Wilk test was performed to assess the normality of continuous variables. The results showed that the data did not follow a normal distribution. Therefore, Spearman correlation analysis was conducted to examine the monotonic associations among family resilience, dyadic coping, and health-related quality of life, and a correlation heatmap was generated (31). Third, LPA was used to identify latent subgroups of family resilience. To evaluate local dependence among the FRAS-C items, we inspected the Spearman correlation matrix. The analysis started with a one-class model and progressively increased the number of classes until no further significant improvement in the fit indices was observed. The fit indices included the Akaike information criterion (AIC), Bayesian information criterion (BIC), sample-size-adjusted BIC (aBIC), entropy, Lo-Mendell-Rubin likelihood ratio test (LMR-LRT), bootstrap likelihood ratio test (BLRT), and class probabilities. Lower values of AIC, BIC, and aBIC, together with a higher entropy value (>0.8), indicated better model fit (17). Subsequently, univariate and multivariate logistic regression analyses were performed to identify factors associated with the LPA-derived profiles, and the results were presented as a forest plot. Bayesian factor analysis was used to test differences in HRQoL scores across different family resilience profiles (32). Fourth, Harman's single-factor test was used to assess common method bias. If the total variance explained by a single factor was less than 40%, common method bias was considered not severe (33). Fifth, differences in dyadic coping and HRQoL among the different family resilience profiles were compared. As a sensitivity analysis, we performed stratified correlation analyses to examine whether the associations between the DCI and HRQoL were consistent across caregiver–patient relationship types. Finally, based on the physical and mental components of HRQoL, hypothesized mediation analyses were conducted using the PROCESS macro (Model 4) to examine the indirect association between LPA-derived family resilience profiles and both physical and mental HRQoL via dyadic coping (34). In these analyses, PCS and MCS were used as the primary outcomes, whereas the overall HRQoL score was used for descriptive and group-comparison analyses, including ANOVA and Bayesian *post-hoc* comparisons, but not in the mediation models. To adequately control for the potential influence of demographic variables on the mediation model, those demographic characteristics that were significantly associated with both family resilience profiles and HRQoL were included as covariates in the final analysis. The significance level was set at α = 0.05. All data analyses were performed using SPSS 27.0, Mplus 8.3, JASP 0.19.3, and R 4.5.2.

## 3 Results

### 3.1 Demographic and clinical characteristics

Among the 572 primary caregivers, the mean age was 52.78 years (SD = 11.93), and 65.0% were female. Univariate analysis revealed that HRQoL was significantly associated with relationship to patient (*P* = 0.001), caregiver educational level (*P* = 0.001), monthly household income (*P* = 0.001), daily care hours (*P* < 0.001), and self-rated health (*P* < 0.001). However, no significant association was found between HRQoL and caregiver gender (*P* = 0.196). Detailed demographic and clinical characteristics are presented in Table 1.

**Table 1:** Demographic and caregiving characteristics and HRQoL among primary caregivers (*N* = 572).

Variables	*n* (%)	HRQoL (M ±SD)	*P*
Caregiver age (years)	–	52.78 ± 11.93	–
15.5-7.4,14242pt**Relationship to patient**			0.001
Spouse	178 (31.1)	34.61 ± 5.98	
Child	320 (55.9)	36.39 ± 5.93	
Other	74 (12.9)	37.30 ± 6.02	
15.5-7.4,14242pt**Caregiver gender**			0.196
Male	200 (35.0)	36.40 ± 5.88	
Female	372 (65.0)	35.72 ± 6.09	
15.5-7.4,14242pt**Caregiver educational level**			0.001
Primary or below	114 (19.9)	34.38 ± 5.70	
Junior high school	211 (36.9)	35.91 ± 6.03	
Senior high school	161 (28.1)	36.41 ± 6.08	
College or above	86 (15.0)	37.30 ± 5.96	
15.5-7.4,14242pt**Monthly household income (RMB)**			0.001
< 3,000	211 (36.9)	34.79 ± 5.63	
3,000–5,000	200 (35.0)	36.09 ± 6.15	
>5,000	161 (28.1)	37.32 ± 6.09	
15.5-7.4,14242pt**Daily care hours**			< 0.001
< 8 h	256 (44.8)	37.30 ± 5.78	
≥8 h	316 (55.2)	34.86 ± 6.01	
15.5-7.4,14242pt**Self-rated health**			< 0.001
Poor	144 (25.2)	34.65 ± 5.35	
Fair	251 (43.9)	35.47 ± 6.15	
Good	177 (30.9)	37.71 ± 5.98	

### 3.2 Correlations among family resilience, dyadic coping, and HRQoL

Harman's single-factor test was used to assess common method bias. All items of the FRAS-C, DCI, and SF-12 were entered into an unrotated principal component factor analysis. The first unrotated factor explained 10.44% of the total variance, which is below the critical value of 40%, indicating that common method bias was not severe. Normality tests showed that the scale scores followed a skewed distribution (*P* < 0.05); therefore, Spearman's rank correlation analysis was used. The results showed that: (1) family resilience was positively correlated with dyadic coping (r = 0.375, *P* < 0.001); (2) family resilience was positively correlated with both PCS (r = 0.354, *P* < 0.001) and MCS (r = 0.351, *P* < 0.001); and (3) dyadic coping was positively correlated with both PCS (r = 0.463, *P* < 0.001) and MCS (r = 0.399, *P* < 0.001). The correlation heatmap is shown in Figure 1.

**Figure 1:**
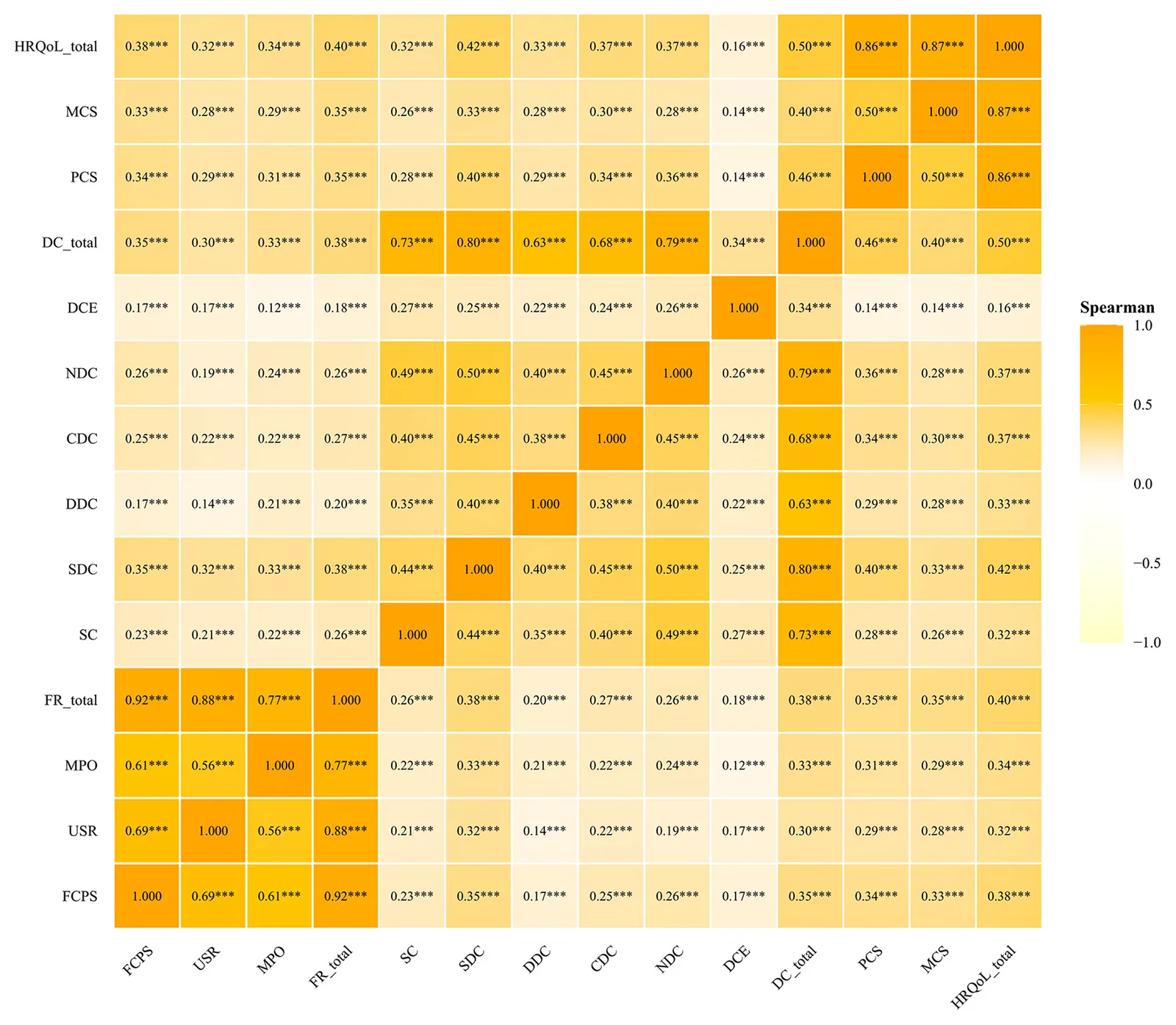
Heatmap of Spearman correlations among family resilience, dyadic coping, and health-related quality of life.

### 3.3 LPA of family resilience

LPA was performed based on the 32 individual items of the FRAS-C. Prior to the analysis, we inspected the Spearman correlation matrix to evaluate local dependence among these items. The maximum absolute correlation was 0.263, and no item pair exceeded 0.4, indicating acceptable local independence. As the number of profiles increased, the AIC, BIC, and aBIC values gradually decreased. Although the four-profile model showed a slightly lower AIC, the three-profile model was ultimately selected as the optimal model based on its lowest BIC and aBIC values, highest entropy (0.815), significant LMR-LRT and BLRT (*P* < 0.001), and superior theoretical interpretability and parsimony. Detailed fit indices are presented in Figure 2A. The three profiles were named the low resilience group (33.9%, Profile 1), the middle resilience group (41.6%, Profile 2), and the high resilience group (24.5%, Profile 3) (Figures 2B, C). Scores on each dimension displayed a distinct gradient across the three profiles (low < middle < high). Both univariate and multivariate analyses revealed that caregiver educational level, monthly household income, daily care hours, self-rated health, and caregiver age were significant factors differentiating the low resilience group from the middle resilience group, as well as the low resilience group from the high resilience group. Results are elaborated in Figure 2D.

**Figure 2:**
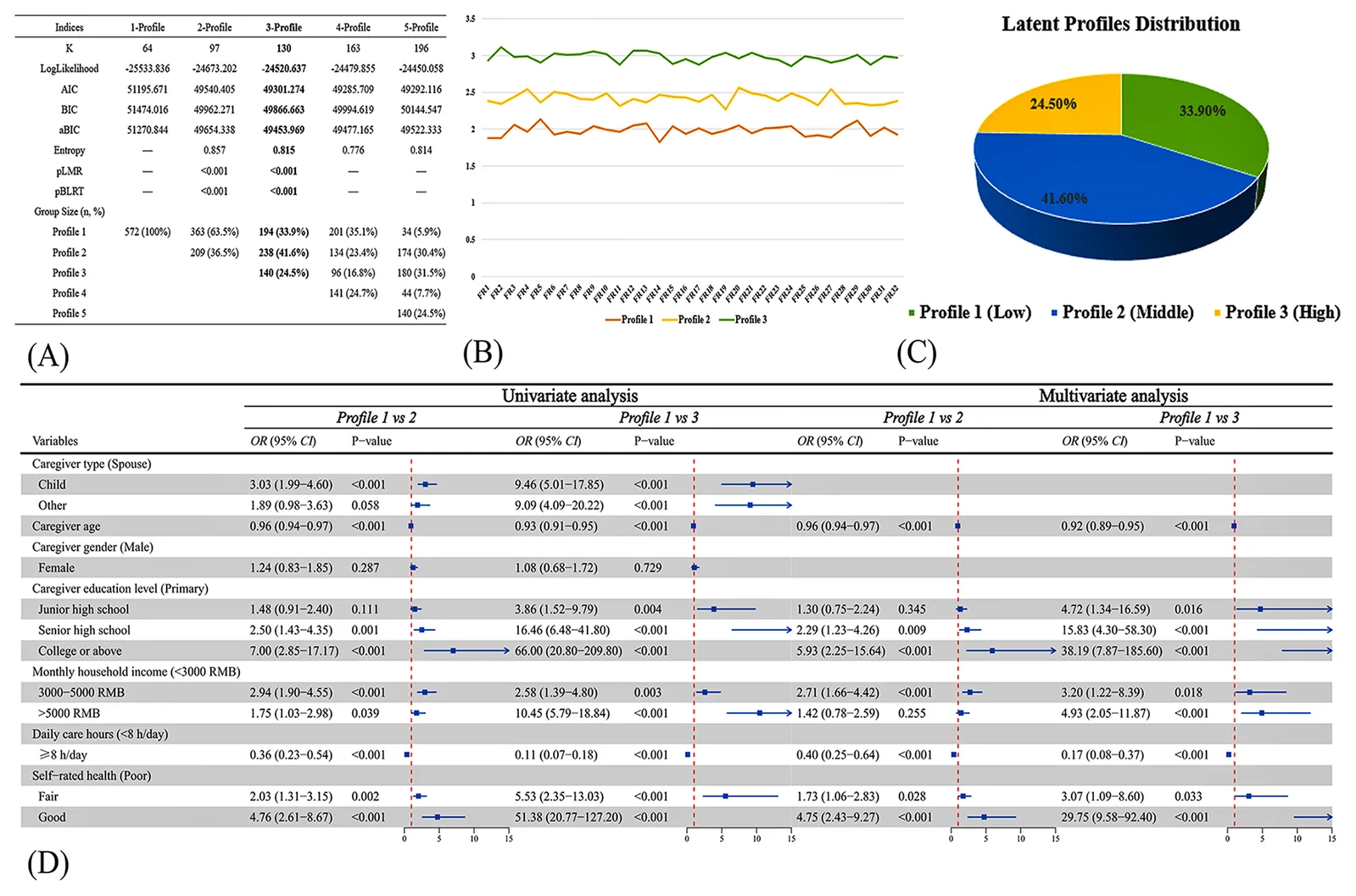
**(A)** Fit indices for the latent profile models. **(B)** The three final latent classes were identified. **(C)** Distribution of the three latent classes. **(D)** Univariate and multivariate logistic regression analyses based on LPA.

### 3.4 Differences in HRQoL scores across LPA-derived profiles

One-way ANOVA was used to compare groups. The results showed that the difference in HRQoL scores among the three groups was statistically significant (*F* = 52.524, *P* < 0.001). Specifically, significant differences were found between the low and middle resilience groups (BF_10_ = 4.482 × 10^6^, d = −0.59), between the low and high resilience groups (BF_10_ = 1.027 × 10^20^, d = −1.19), and between the middle and high resilience groups (BF_10_ = 5,377.252, d = −0.51). Detailed results are presented in Figure 3.

**Figure 3:**
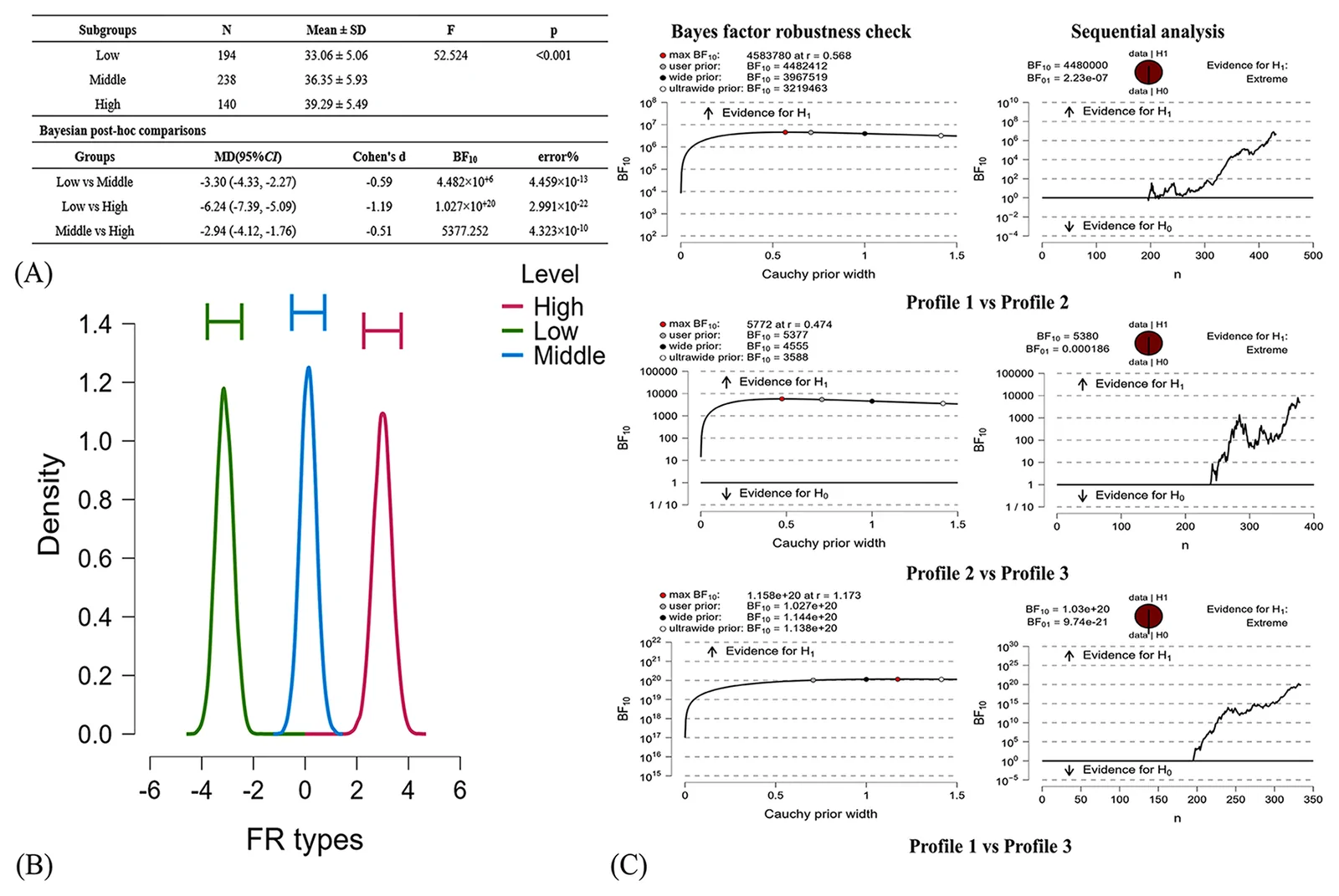
ANOVA comparison of HRQoL scores across family resilience profiles and *post-hoc* comparison by Bayesian factor analysis. **(A)** LPA-based differences in HRQoL scores were estimated by Bayesian factor. **(B)** ANOVA model averaged posterior distribution. **(C)** Inferential plots for Bayesian factor analysis.

### 3.5 Differences in dyadic coping and HRQoL scores across LPA-derived profiles

The three resilience profiles differed significantly in both dyadic coping and HRQoL (all *P* < 0.001). *Post-hoc* pairwise comparisons (LSD) revealed that, except for delegated dyadic coping and evaluation of dyadic coping, where the differences between the middle and high resilience groups were not statistically significant (*P* = 0.312 and *P* = 0.060, respectively), scores for all other variables followed an increasing trend from the low resilience group to the middle resilience group to the high resilience group (all *P* < 0.001). The results are illustrated in Figure 4A.

**Figure 4:**
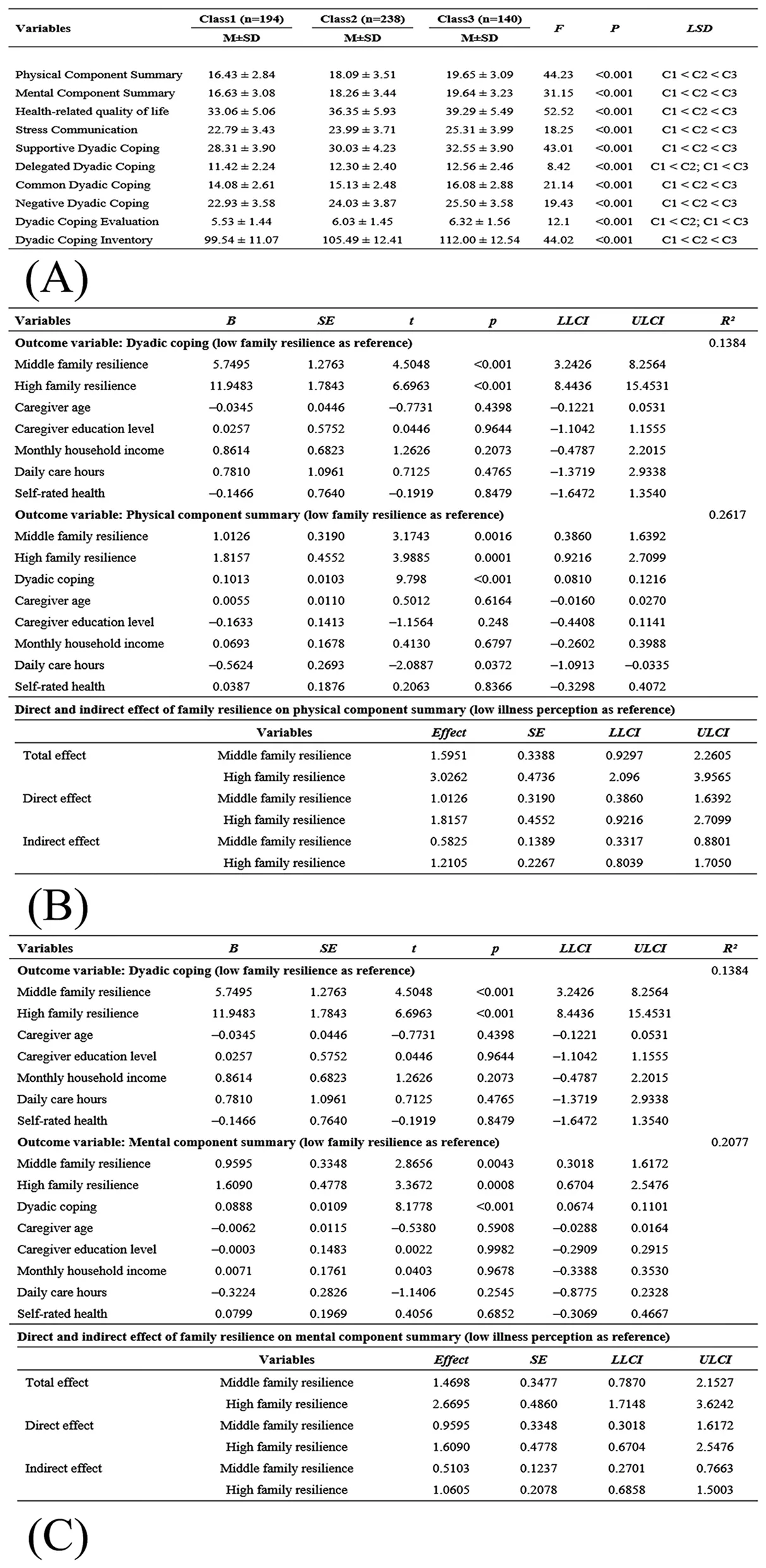
**(A)** Differences in dyadic coping and health-related quality of life scores across family resilience profiles. **(B)** Indirect association between family resilience profiles and Physical Component Summary via dyadic coping. **(C)** Indirect association between family resilience profiles and Mental Component Summary via dyadic coping.

### 3.6 Sensitivity analysis: consistency of DCI–HRQoL associations across caregiver subgroups

As a sensitivity analysis, we performed stratified correlation analyses to examine whether the associations between the DCI and HRQoL were consistent across caregiver–patient relationship types. As shown in Table 2, DCI total scores were significantly and positively correlated with PCS, MCS, and overall SF-12 scores across all three subgroups: spousal caregivers (*n* = 178), child caregivers (*n* = 320), and other caregivers (*n* = 74), with all *p*-values < 0.001. The correlation coefficients ranged from 0.390 to 0.489 in spousal caregivers, 0.371 to 0.472 in child caregivers, and 0.453 to 0.548 in other caregivers, demonstrating broadly consistent effect sizes across subgroups.

**Table 2:** Sensitivity analysis of DCI–HRQoL correlations by caregiver relationship type.

Subgroup	*n*	PCS	MCS	SF12_Total
Spouse	178	r = 0.463, *P* < 0.001	r = 0.390, *P* < 0.001	r = 0.489, *P* < 0.001
Child	320	r = 0.456, *P* < 0.001	r = 0.371, *P* < 0.001	r = 0.472, *P* < 0.001
Other	74	r = 0.453, *P* < 0.001	r = 0.475, *P* < 0.001	r = 0.548, *P* < 0.001

Spearman's rank correlation coefficients were used for all analyses.

### 3.7 Indirect associations of dyadic coping between family resilience and HRQoL

Using the low resilience group as the reference category, hypothesized mediation models were constructed with dyadic coping as the intervening variable and PCS and MCS as outcome variables, respectively. Caregiver age, caregiver educational level, monthly household income, daily care hours, and self-rated health were included as covariates. The bias-corrected bootstrap method (5,000 resamples) was used to calculate the 95% confidence intervals for the indirect effects.

The results showed: (1) For PCS: In the middle resilience group, the total effect was 1.5951 (95% *CI*: 0.9297–2.2605), the direct effect was 1.0126 (95% *CI*: 0.3860–1.6392), and the indirect effect was 0.5825 (95% *CI*: 0.3317–0.8801). In the high resilience group, the total effect was 3.0262 (95% *CI*: 2.0960–3.9565), the direct effect was 1.8157 (95% *CI*: 0.9216–2.7099), and the indirect effect was 1.2105 (95% *CI*: 0.8039–1.7050). (2) For MCS: In the middle resilience group, the total effect was 1.4698 (95% *CI*: 0.7870–2.1527), the direct effect was 0.9595 (95% *CI*: 0.3018–1.6172), and the indirect effect was 0.5103 (95% *CI*: 0.2701–0.7663). In the high resilience group, the total effect was 2.6695 (95% *CI*: 1.7148–3.6242), the direct effect was 1.6090 (95% *CI*: 0.6704–2.5476), and the indirect effect was 1.0605 (95% *CI*: 0.6858–1.5003). These results indicate statistically significant indirect associations through dyadic coping between family resilience profiles and caregiver HRQoL. The specific indirect association pathways are shown in Figures 4B, C, and Figure 5.

**Figure 5:**
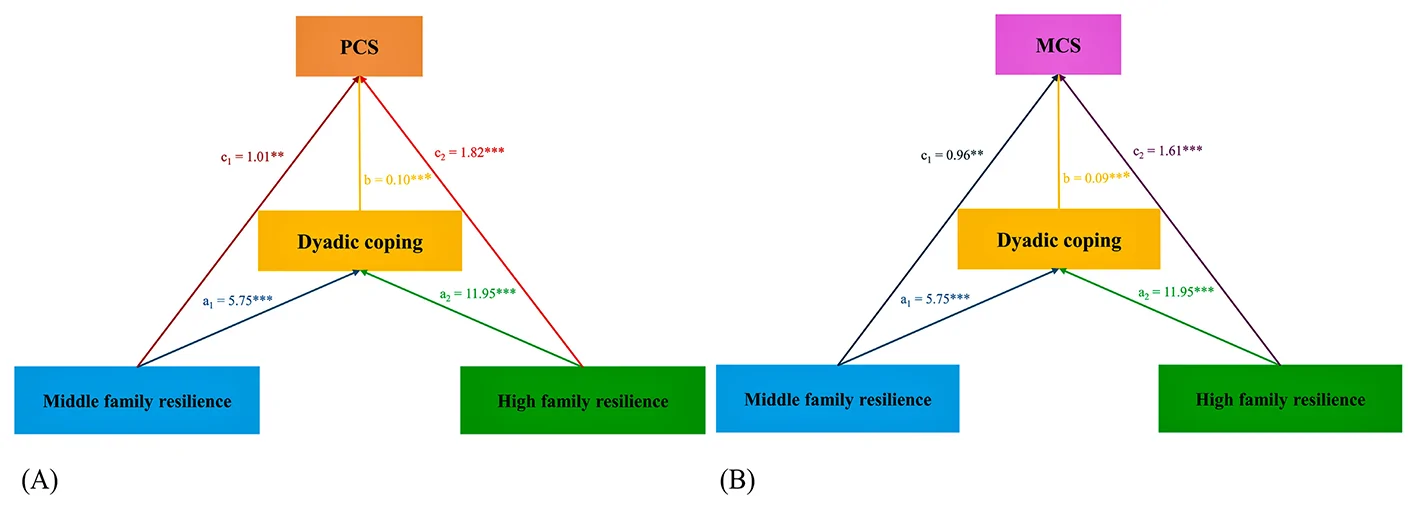
**(A)** Indirect association between family resilience profiles and Physical Component Summary via dyadic coping. **(B)** Indirect association between family resilience profiles and Mental Component Summary via dyadic coping. *** *P* < 0.001; Low family resilience as reference; Covariates: caregiver age, caregiver education level, monthly household income, daily care hours, self-rated health.

## 4 Discussion

This study is the first to reveal the heterogeneous characteristics of family resilience among primary caregivers of hospitalized older adults with multimorbidity in Xinjiang, and to examine whether the findings were consistent with a hypothesized mediation model in which dyadic coping was indirectly associated with the relationship between family resilience and caregiver HRQoL (35, 36). The main findings are: caregivers could be classified into three family resilience profiles (low, middle, and high); caregiver educational level, monthly household income, daily care hours, self-rated health, and caregiver age were independent factors distinguishing these profiles; and dyadic coping was indirectly associated with the relationship between family resilience profiles and both physical and mental HRQoL. These results highlight the importance of shifting clinical focus from individual coping to family system resources, and of integrating dyadic coping interventions into stratified psychosocial support strategies for caregivers (20, 37). It is important to note that these profiles primarily differed in the overall level of family resilience, representing quantitative rather than qualitative differences. This suggests that family resilience among these caregivers varies in degree rather than in kind. Therefore, interventions could focus on enhancing the overall level of family resilience.

Firstly, we identified three latent profiles of family resilience among primary caregivers of hospitalized older adults with multimorbidity: low resilience (33.9%), middle resilience (41.6%), and high resilience (24.5%). This distribution suggests that a considerable proportion of caregivers have low family resilience and may find it difficult to cope effectively with the multiple challenges of caring for a person with multimorbidity (3, 38). Family resilience refers to the ability of a family system to maintain function and adapt in the face of adversity; it is shaped by factors such as family communication, resource utilization, and positive beliefs (10, 11). Caring for a person with multimorbidity involves complex symptom management, frequent medical decisions, and heavy financial burdens (3, 4). These challenges may weaken caregivers' family communication and problem-solving abilities, reduce their efficiency in using social resources, and undermine their positive beliefs (16). Our study found that family resilience was positively correlated with dyadic coping and caregiver HRQoL, suggesting that caregivers with higher family resilience may be more likely to adopt positive dyadic coping strategies and may also have better physical and mental health (39). By using latent profile analysis, this study went beyond traditional variable-centered approaches and provided a more refined identification of heterogeneous caregiver subgroups, laying an empirical foundation for stratified and targeted psychosocial interventions (18, 38).

Demographic and caregiving-related characteristics significantly influenced family resilience profiles. Caregivers with higher educational attainment exhibited greater resilience, likely due to enhanced health literacy and resource-seeking capabilities (40). Additionally, those with higher monthly household incomes demonstrated increased resilience, aligning with prior research indicating that economically advantaged families typically possess greater stress resistance and more extensive social support resources (20, 41). Conversely, caregivers who provided daily care for 8 h or more reported significantly lower resilience, suggesting that prolonged caregiving depletes psychological resources (42, 43). Self-rated health emerged as the strongest predictor of family resilience, indicating a bidirectional relationship between the caregiver's health and the family's adaptive capacity (44). This study also revealed that older caregivers exhibited lower levels of family resilience. Descriptive statistics indicated that the mean age of caregivers in the high resilience group was significantly lower than that of their counterparts in the low resilience group. Both univariate and multivariate regression analyses consistently corroborated this finding, demonstrating a negative association between caregiver age and family resilience. A plausible explanation is that younger caregivers typically possess more physical energy, enhanced psychological resilience, and greater social adaptability, enabling them to navigate caregiving challenges more effectively. This finding aligns with previous studies, underscoring the significance of age as a factor influencing caregiver resilience (44, 45). In the multivariate model, caregiver type was no longer a significant factor, suggesting that the previously noted “higher resilience in child caregivers” is primarily accounted for by variations in age, education, and income, rather than by the caregiver-child relationship itself (46).

Secondly, our study found that dyadic coping was indirectly associated with the relationship between family resilience profiles and caregiver HRQoL, providing evidence consistent with Hypothesis 2. Specifically, among caregivers with middle and high resilience, dyadic coping may be one pathway linking higher levels of family resilience to better physical and mental HRQoL (i.e., stress communication and joint coping behaviors between the caregiver and the patient) (20, 47). This finding is consistent with Bodenmann's systemic transactional model, which proposes that dyadic coping reframes stress from “my burden” to “our problem”, thereby promoting mutual support and reducing feelings of isolation (13, 14). In our study, scores on most dimensions of dyadic coping (except delegated dyadic coping and evaluation of dyadic coping, where the difference between the middle and high resilience groups was not significant) showed an increasing trend from the low resilience group to the middle resilience group to the high resilience group. These findings suggest that dyadic coping may be one pathway linking family resilience to better caregiver health. Therefore, clinical interventions should not only focus on individual psychological adjustment but also strive to improve dyadic coping between caregivers and patients, for example, by facilitating stress communication through family meetings and providing joint problem-solving training (39, 48).

This study compared differences in HRQoL across resilience profiles using both frequentist statistics and Bayesian factor analysis. Bayesian analysis provided complementary evidence to quantify the strength of the findings (49). Unlike traditional statistics that only report *p*-values, the Bayes factor (BF10) quantifies the evidence supporting the alternative hypothesis (i.e., that differences exist between groups) relative to the null hypothesis, and is less strictly dependent on the assumption of normality. According to Quintana and Williams, a BF10 greater than 10 indicates strong evidence, and greater than 100 indicates very strong evidence (32, 49). In this study, the BF10 for the comparison between the low and high resilience groups was 1.027 × 10^20^, far exceeding 100, indicating extremely strong evidence for group differences. It is worth noting that the Bayesian factor analysis showed significant differences in HRQoL across resilience profiles, which may be partly attributable to the specificity of our sample—primary caregivers of hospitalized older adults with multimorbidity in tertiary hospitals in Xinjiang share high disease burdens and similar health-seeking cultural backgrounds (49, 50). This homogeneity may lead to limited within-group variation and magnified between-group differences. Future studies should validate these findings among broader and more diverse samples of community-dwelling older caregivers (38, 51).

In summary, this study clarifies the heterogeneous nature of family resilience among primary caregivers of hospitalized older adults with multimorbidity, and identifies dyadic coping as being indirectly associated with the relationship between family resilience profiles and HRQoL (3). These findings have important implications for clinical practice. When assessing and intervening in caregiver health, attention should be paid to both family resilience and dyadic coping. For caregivers with middle or low resilience profiles, intervention strategies should include measures aimed at strengthening family resilience and promoting dyadic coping, such as family support skills training, stress communication training, and joint problem-solving workshops (20, 39). By strengthening the family system and dyadic interaction mechanisms, we can more comprehensively and sustainably improve caregivers' mental health and quality of life (10, 11). Future research should further employ longitudinal or interventional designs to validate the causal direction of these findings and explore the stability of these indirect associations across different cultural contexts (52).

## 5 Limitations

This study presents several limitations. First, the sample was obtained from three tertiary hospitals in Xinjiang. Given the similar medical environments, significant disease burdens, and comparable age and cultural characteristics, this population exhibited considerable homogeneity in psychological and behavioral responses (50). Notably, caregivers in the high resilience group demonstrated relatively high HRQoL scores; however, within-group variation was limited. These findings require further validation in larger and more diverse samples of community-dwelling older caregivers (51). Additionally, future research should incorporate qualitative interviews to achieve a deeper understanding of the nuanced differences in the psychological status of primary caregivers who provide long-term care for older adults with multimorbidity.

Second, this study included only seven demographic and caregiving-related variables (age, sex, relationship to patient, educational level, monthly household income, daily care hours, and self-rated health). Other potential factors that may influence caregiver HRQoL, such as marital status, employment status, living with the patient or not, and place of residence, were not measured (53). The absence of these confounding variables may have affected the results. Future research should incorporate a broader array of demographic and sociocultural variables to enhance the robustness of our conclusions (38).

Third, this study used a cross-sectional design. Although mediation models were tested to estimate indirect associations among variables, causal inferences about the relationships between family resilience, dyadic coping, and HRQoL cannot be made. Future research should adopt longitudinal or interventional designs to further clarify these relationships (52).

Fourth, this study was conducted only in Xinjiang. Therefore, factors such as diversity in ethnic culture and family structure, as well as accessibility of medical resources, may limit the generalizability of the findings to other populations and regions (50). Multi-center studies covering broader geographical areas are needed to enhance the generalizability of the results.

Fifth, although common method bias was assessed, all key variables were obtained through self-report questionnaires, so some bias may still exist. Future studies are encouraged to include appropriate objective indicators (e.g., physiological stress markers or observational assessments) as complementary measures (54, 55).

Sixth, this study included only caregivers as survey respondents and did not simultaneously collect patient-level clinical indicators, such as the number of chronic conditions and functional status scores. Consequently, we could not examine whether patients‘ disease severity exerted confounding effects on caregivers' family resilience, dyadic coping, or HRQoL (22). Future studies may consider collecting patient burden data alongside caregiver reports to better control for potential confounders.

Seventh, although the DCI was originally validated primarily in Chinese couples, our stratified analyses showed consistent associations between dyadic coping and HRQoL across spousal, child, and other caregiver subgroups (28). However, the possibility of measurement heterogeneity across different caregiver–patient relationship types cannot be entirely ruled out, and future studies should further validate the DCI in non-spousal caregiver populations.

Finally, dyadic coping was assessed solely from the caregiver's perspective. Although caregiver-perceived dyadic coping is a commonly used and valid measure, single-informant reports cannot fully capture the interactive process of dyadic coping between caregivers and patients (56, 57). Future research could collect data from both dyads to gain a more comprehensive picture of the dynamic nature of dyadic coping.

## 6 Conclusions

This study identified significant heterogeneity in family resilience among primary caregivers of hospitalized older adults with multimorbidity, classifying them into three latent subgroups: low, middle, and high resilience. Dyadic coping was indirectly associated with the relationship between family resilience profiles and caregiver HRQoL, indicating an indirect relationship among these three variables. These findings offer preliminary support for individualized psychological intervention strategies for caregivers with different family resilience profiles. It should be noted that, due to the cross-sectional design of this study, causal relationships among the variables cannot be established. Future research should consider using longitudinal or interventional designs to test these causal pathways, and should expand sample sizes and geographical scope to enhance the external validity of the results.

## Data Availability

The original contributions presented in the study are included in the article/supplementary material, further inquiries can be directed to the corresponding author.
